# Nursing Staff in a Large Hospital System Underutilize Insurance-Based Mental Health Services

**DOI:** 10.3390/healthcare12121188

**Published:** 2024-06-13

**Authors:** Chandra L. Bautista, Katelynn A. Bourassa, Namrata N. Vasquez, Madeleine Desrochers, Nicole Bartek, Alok Madan

**Affiliations:** 1 Department of Psychiatry & Behavioral Health, Houston Methodist, Houston, TX 77030, USA; cbautista2@houstonmethodist.org (C.L.B.); nnvasquez@houstonmethodist.org (N.N.V.); mdesrochers@houstonmethodist.org (M.D.); nbartek@houstonmethodist.org (N.B.); amadan@houstonmethodist.org (A.M.); 2 Houston Methodist Academic Institute, Houston, TX 77030, USA; 3 Houston Methodist Research Institute, Houston, TX 77030, USA; 4 Department of Psychiatry, Weill Cornell Medical College, New York, NY 10065, USA

**Keywords:** nursing, mental healthcare, burnout, service utilization

## Abstract

Nurses are at high risk of burnout and subsequent mental health concerns due to problems with overstaffing, immense workload volume, and personal health risks associated with the job. Effective mental health treatments are available but potential barriers to receiving care may prevent nurses from benefiting. The Emotional Health and Well-Being Clinic (EHWC) at Houston Methodist is an outpatient mental health clinic offering therapy and medication management services for employees and employee dependents of our institution. The EHWC is uniquely positioned to observe how nurses utilize mental health services and to address barriers to effective care for this vital group of healthcare professionals. This paper provides descriptive data on the utilization of mental health services by nurses in the EHWC and a discussion of possible challenges faced by this group when seeking care. Based on these data, we propose potential solutions to ensure that nurses can achieve maximum benefit from outpatient mental health services.

## 1. Introduction

Burnout, a state of emotional exhaustion, perceived low achievement, and negative attitudes brought on by chronic work stress, is a pervasive, global crisis among healthcare workers [1]. Burnout is associated with poor health outcomes for healthcare workers, poor patient care, medical errors, and high organizational costs related to absenteeism and turnover [2]. Nurses are known to be at particularly high risk of burnout [3]. For example, a recent survey of physicians and nurses employed by Magnet hospitals in the United States reported that a greater proportion of nurses than physicians endorsed burnout symptoms [4]. Rates of burnout have increased in the COVID-19 era [5]. More broadly, nurses consistently experience high rates of depression [6,7,8], trauma-associated stress and anxiety [9], and suicidality [10]. Contributing factors to nurse burnout and psychological distress include depleted workforce, high patient volumes, personal health risk, and exposure to traumatic and stressful events [11]. Mental health interventions are available and effective [12], and include interventions specifically targeting burnout in healthcare workers [13]. While the utilization of these interventions is unknown for this group, it is likely to be low, given known barriers that impact the accessibility of care.

Despite the growing mental health need among nurses [4,5,6,7,8,9,10], rates of help-seeking are low. For example, a 2022 national survey suggested that only 35% of nurses with mental health needs sought care [14]. Nurses may not only experience traditional barriers to care experienced by the general population of adults with mental health concerns (e.g., leave from work, cost, childcare, transportation), but unique barriers associated with their profession. For example, shift work (e.g., three 12 h shifts per week, night shift) may complicate scheduling mental health visits, which are typically offered during standard business hours. Furthermore, stigma and concerns about the impact of disclosing mental health status on licensure and employment are common barriers to help-seeking among nurses and other healthcare professionals [14,15].

The Emotional Health and Well-Being Clinic (EHWC) is an outpatient mental health clinic that serves approximately 30,000 employees of a major hospital system and their healthcare dependents. To our knowledge, the EHWC is among the first, if not the only, clinic of this kind in the country. The clinic was founded in 2021 and is staffed by a multidisciplinary team of mental health professionals offering medication management and individual, group, and couples therapy. The clinic is located in the system’s flagship hospital and offers free (no co-pay) services with convenient, flexible in-clinic or virtual appointments for those on the insurance plan. The EHWC was designed to remove the common barriers to accessing mental health services, such as location, availability, cost, and support from employers (e.g., approved leave for appointments, coordination of back-up staffing coverage) to attend appointments.

Little is known about the mental healthcare utilization patterns of nurses. The purpose of this paper is to examine data collected over the first two years of clinic operation to describe the feasibility and accessibility of this resource for nurses, who may face unique challenges when trying to engage in mental healthcare (e.g., stigma, scheduling). Specifically, this paper contributes descriptive data about the services used and courses of care for nurses who received services in a clinic designed to support healthcare workers. Exploratory analyses were conducted to examine the potential impact of demographic characteristics (i.e., race, age) associated with disparities in mental healthcare engagement [16] as well as of work stress on care engagement in the clinic. This information may contribute to the development of more tailored services for this population.

## 2. Materials and Methods

### 2.1. Participants and Setting

Data were collected from patients enrolled in the outpatient mental health clinic of a large hospital system in the southwestern United States from fall 2021 to fall 2023. Patients were eligible for services in the clinic if they were (1) an employee of the hospital system or a dependent on the insurance plan of an employee of the hospital system, (2) were at least 18 years of age, and (3) were able to provide informed consent for care. Patients were included in this study if they (1) met all of the inclusion and none of the exclusion criteria to receive services in the clinic, (2) were enrolled in the clinic during the identified timeframe, (3) were employed by our institution, and (4) reported their profession as nursing. Of the 639 individuals who requested mental health services during the study period, 130 patients (20.30%) were identified as nurses and were included in this study.

### 2.2. Procedure

This naturalistic, observational study involved secondary analysis of treatment utilization data collected during the first two years of the EHWC’s operation. This study was conducted in accordance with the Declaration of Helsinki and approved by the Institutional Review Board of Houston Methodist. Informed written consent was waived as this study involved only secondary data analysis of data collected as standard of care. A list of patients served in the study timeframe was maintained by clinic staff. The clinic operations team provided the research team with utilization data for each patient. This information was compared with the electronic medical record. Abstracted data were de-identified and collated into a single electronic document and stored on a secured shared drive accessible only to study staff.

Data coders were the primary authors, who reviewed clinic records to identify all patients who enrolled in the clinic during the study period. Patients who reported their profession as nursing were selected for this study. Data, including demographic information (i.e., age, race, ethnicity, gender identity, sexual orientation, profession), diagnoses, and information related to work stress as a contributor to distress, were abstracted from the medical record. The data abstraction and coding plan was discussed prior to record review. Data coders met weekly to discuss and update the coding process, as needed, to ensure reliability.

Psychiatric diagnoses were categorized following DSM-5 classifications (e.g., depressive disorders, anxiety disorders). An “other diagnosis” category was created to capture presenting concerns not reflected by these categories (e.g., life stressors, mood disorder). A diagnostic category was coded as present if the diagnosis was listed in the “visit diagnosis” for the appointment encounter and/or in the documentation for the visit as a current presenting concern. Historical diagnoses were not included. If patients had more than one diagnosis listed, all current diagnoses were included.

Work stress was coded as present if patients indicated that some element at work was causing or contributing to their distress. This variable reflects spontaneous reports of work stress only and was not included as standard language in the intake assessment procedure.

Service utilization data were also abstracted from the medical record, including type of encounter (e.g., physician visit) and whether the encounter was completed, missed, or cancelled. To best capture the services in the clinic, the following categories were used: (1) medication management, (2) individual psychotherapy, (3) group psychotherapy, and (4) couples counseling. In the clinic, prescribers can serve in both a medication management and/or psychotherapy role; however, it was not always possible to determine the type of service provided by prescribers through chart review. As such, any visit with a prescriber was coded as medication management. Furthermore, creative arts therapists (e.g., music therapy, art therapy) are employed in the clinic and as it was not always possible to determine from the chart review whether an encounter involved creative arts therapy or another individual therapy modality, all services provided by creative arts therapists were grouped into the individual psychotherapy category. After coding services, the authors tallied the number of scheduled, completed, cancelled, and no-showed visits for each patient for each service type.

### 2.3. Data Analysis

Data were analyzed using SPSS version 29 [17]. Descriptive statistics were used to characterize the sample, including: all individuals who requested services in the clinic, patients who were seen in the clinic, and the subset of patients who participated in each treatment modality. One patient with incomplete appointment attendance data was excluded from all analyses. Descriptive statistics were also used to characterize treatment participation.

Given the range of treatment sessions scheduled and completed, a “percentage of sessions attended” variable was computed to better capture the average appointment attendance for each patient across each of the treatment modalities. To explore whether the level of service utilization affected appointment attendance, percentiles were used as cut points for the number of sessions scheduled to create three groups: low, moderate, and high service utilizers. A series of one-way ANOVAs were used to explore the effect of service utilization on appointment attendance, as defined by the percentage of sessions attended. Given small sample sizes, low and moderate users of couples therapy were combined into a single category. To address heteroscedasticity, Welch’s ANOVA with Games–Howell correction for multiple comparisons was used for therapy and medication service analyses; ANOVA with Tukey correction was applied for all other service types. To account for unequal sample sizes, race was recoded as a three-level categorical variable: (1) Black, Indigenous, Hispanic, Asian, and other people of color, (2) white, and (3) declined to answer. A one-way ANOVA explored the effect of race on appointment attendance. A series of separate independent t-tests were conducted to explore the effect of work stress and age on appointment attendance for each treatment modality. For the age analyses, the cut point was identified as the age at the 50th percentile.

## 3. Results

One hundred thirty nurses requested the EHWC’s services during the study period. One patient with incomplete treatment utilization data was excluded from analyses. Interested nurses (N *=* 129) were an average age of 38.47 years (SD 9.72), and the majority identified as white (n = 75, 58.1%), non-Hispanic/Latino (n = 87, 67.4%), and female (n = 110, 85.3%). See Table 1 for demographic information of the overall sample. Of the 129 individuals who expressed interest in services, 11 (8.5%) never scheduled an appointment in the clinic, 4 (3.1%) scheduled an appointment and never attended, and 114 (88.4%) were seen for at least one appointment. The patients seen in the clinic were an average of 38.66 years of age (SD 9.93), white (n = 71, 62.3%), non-Hispanic/Latino (n = 81, 71.1%), and female (n = 100, 87.7%). Fifty-five patients (48.3%) used more than one treatment service in the clinic. A near majority of patients (n = 52, 45.6%) used a combination of medication management and psychotherapy services. Most patients received one psychiatric diagnosis (n = 63, 55.3%). Depressive disorders (n = 55, 48.2%) and anxiety disorders (n = 50, 43.9%) were the most common diagnoses (see Table 2). More than half of patients (n = 69, 53.5%) reported work stress as a contributing factor to their distress. 

Clinic utilization data are reported separately for each treatment modality in Table 3 below.

### 3.1. Individual Psychotherapy

Eighty-eight patients were scheduled for at least one individual psychotherapy session. The average attendance rate was 76.6% (SD 21.96). Appointment attendance did not significantly differ among low to high service utilizers, *F*(2, 53.85) = 0.02, *p* = 0.98, race *F*(1, 86) = 0.39, *p* = 0.53, by presence or absence of work stress *t*(86) = 0.23, *p* = 0.82, or by age, *t*(85) = 0.16, *p* = 0.67.

### 3.2. Medication Management

Seventy-six patients were scheduled for at least one medication management appointment. The average attendance rate was 73.5% (SD 19.46). Appointment attendance did not significantly differ among low to high service utilizers, *F*(2, 43.58) = 0.30, *p* = 0.75, race, *F*(1, 74) = 0.52, *p* = 0.82, by presence or absence of work stress, *t*(74) = −1.68, *p* = 0.10, or by age *t*(74) = 0.94, *p* = 0.61.

### 3.3. Group Psychotherapy

Twelve patients were scheduled for group psychotherapy. The average rate of attendance was 47.8% (SD 25.04). Appointment attendance did not significantly differ among low to high service utilizers *F*(2, 9) = 0.05, *p* = 0.96, by presence or absence of work stress *t*(10) = 1.32, *p* = 0.22, or by age *t*(10) = 1.15, *p* = 0.63. Appointment attendance differed by race, such that Black, Indigenous, Hispanic, Asian, and other people of color attended, on average, a lower percentage of group sessions (M 30.77, *SD* 19.69) than white patients (M 60.06, SD 21.77), *F*(1, 10) = 5.69, *p* = 0.04.

### 3.4. Couples Counseling

Eight patients were scheduled for couples counseling. The average rate of attendance was 85.3% (SD 16.25). Appointment attendance did not significantly differ between low + moderate to high service utilizers, *F*(1, 6) = 0.56, *p* = 0.48, race *F*(1, 6) = 0.10, *p* = 0.92, by presence or absence of work stress *t*(6) = −0.51, *p* = 0.63, or by age *t*(6) = 0.10, *p* = 0.74.

## 4. Discussion

Nurses are at high risk for burnout and other forms of psychiatric distress [4,5,6,7,8,9,10] and may face barriers to utilizing mental health services. To our knowledge, the EHWC is among the first, if not the first, clinic of its kind in the United States. It was designed to meet the mental health needs of nurses and other healthcare workers in a large hospital system and to remove traditional barriers to care (e.g., managed care limitations, scheduling challenges). Our institution employs approximately 8000 nurses, which is 26.7% of the workforce. Nurses comprised 20.30% of the patient population in the EHWC.

Extrapolating from epidemiological [14] and local data, findings from this study provide support that nurses markedly underutilize mental health services. National surveys suggest that nurses experience mental health concerns at higher rates than their peers in other professions, with one survey of over 2500 nurses demonstrating high rates of self-reported burnout (75%), feelings of depression (64%), and trauma-related concerns (50%) [14]. Considering that depressive disorders were the most frequent presenting concern in the EHWC and the high rates of depression among nurses nationally [14], nearly 1800 nurses in our institution’s hospital system would be expected to seek mental healthcare each year. Approximately 7% (*N* = 130) of the expected number of nurses sought the services available to them, and a substantial proportion of nurses (11.5%) who enrolled in the clinic never attended the initial appointment.

Once enrolled in care in the EHWC, however, the average attendance rate for individual psychotherapy and medication management was roughly 75%. While we are not aware of other studies examining rates of mental healthcare utilization among this population, the attendance rates of nurses in this study mirror the attendance rates found in studies of mental health treatment engagement in the general population [18]. With the exception of group psychotherapy, service utilization, demographics, and work stress did not impact appointment attendance. The uniform attendance rates suggest that nurses across all of the sociodemographic groups and occupational factors explored in this study faced similar challenges to engaging in services. A potential exception is that patients who identified as Black, Indigenous, Hispanic, Asian, and other people of color attended a lower percentage of group psychotherapy appointments on average than white patients. However, given the small sample size, this conclusion cannot be reliably drawn from these data. Further research is needed to explore potential disparities in care.

There are several well-established barriers to care (i.e., stigma, concern about impact to licensure [14,15]) that may prevent nurses from engaging in mental health services offered at their place of work. Furthermore, nurses tend to face demanding and unpredictable work schedules (e.g., three 12 h shifts per week, occurring on different days or nights), which may account for some of the difficulty with initiating and/or engaging in a standard course of weekly therapy sessions. Additionally, more than half of nurses spontaneously reported work-related stress as a contributor to their overall distress. This is likely a low estimate given that it is based on observational data (i.e., chart extraction) only and was not directly measured for all patients. The EHWC has tried to counteract potential work-related barriers by offering flexible hours and virtual appointments. Based on the patterns of treatment utilization in the EHWC, it is possible that the clinic’s design reduces barriers to staying in care once engaged but does not adequately address barriers to accessing care. As the EHWC is among the first, if not the only, clinic of its kind in the United States, there is a dearth of comparative treatment utilization data from other similar programs. Additional research is needed to explore whether different engagement strategies are needed to accommodate initiation of care and consistent engagement in care.

It is likely that new innovations are needed to enhance the accessibility and acceptability of mental healthcare for nurses. Anecdotally, nurses who participated in treatment in the clinic voiced that scheduling challenges (e.g., day vs. night shift, unexpected shifts) were a barrier for them to consistently engage in treatment. One solution currently in development in the EHWC is a one-day, intensive group intervention that would allow nurses to gain psychoeducation and applicable skills quickly. Related one-day interventions have demonstrated effectiveness in other treatment-seeking populations who experience barriers to care [19]. After the one-day intervention, nurses may choose to engage in flexible follow-up appointments to learn more about applying the information and skills to their specific needs. These follow-up appointments would be scheduled as needed, rather than the standard weekly or biweekly appointments routinely offered in outpatient mental health settings. Further, providing psychoeducation and skills-based outreach services to nurses in their work environment may help overcome mental health stigma [20,21], as all staff would be invited to learn about mental health and support one another. This care model may be both efficient, cost-effective, and adaptable to individual healthcare organizations interested in providing mental health programming for their staff. Additionally, research on disparities in access to care and unique treatment preferences is needed to tailor services to a culturally diverse workforce. To address barriers to mental healthcare among nurses, healthcare organizations may consider offering flexible, affordable, and adaptable services with care options available through multiple modalities, such as outreach to staff on work units, in-house clinic-based services, and covered resources in the community. Given the increasing demands on nursing services across healthcare settings, proactively addressing their mental health and well-being are essential for a safe and effective healthcare system.

The lessons learned from the literature and the EHWC may contribute to the development of more effective models of mental health service delivery across healthcare organizations in the United States. Leaders of healthcare organizations may consider implementing the following supports (see Table 4) for their nursing and healthcare staff:

### Limitations

This study relied on observational data and did not specifically collect information on reasons for missed appointments or treatment dropout. This precluded the ability to determine whether barriers to care commonly identified in the literature are experienced by nursing staff at this organization. Second, data on clinical outcomes as well as some demographic variables were not consistently available in the medical record. Therefore, it was not possible to consider the impact of symptom presentation and severity or occupational variables (e.g., years of practice, department/unit) on treatment utilization. The clinic continues to work toward a streamlined, scalable process for measurement-based care to guide treatment and track patient outcomes. Providers have used self-report questionnaires to guide treatment since the clinic’s inception, but logistical challenges to data collection and management have prevented the use of those data on an aggregate level. A digital solution for measurement-based care is in progress and will allow for ongoing analysis of treatment effectiveness and variables that may predict patient outcomes. Third, while diagnoses were collected to provide an overall clinical picture, they may not fully capture all presenting concerns that were the focus of treatment (e.g., insomnia). Fourth, the sample sizes of those participating in group psychotherapy and couples counseling were small. Findings that nurses of color completed fewer sessions of group psychotherapy than white nurses should be interpreted with caution. Finally, the data were collected from one healthcare organization and findings may not be generalizable to nurses in other healthcare organizations across the country.

Future research should directly assess barriers to mental healthcare experienced by nurses to support the development of more accessible interventions, including studies testing the potential solutions proposed above. Additional research is also needed to explore the impact of demographic, occupational, and clinical factors on treatment utilization. These data may allow for a more nuanced understanding of who can access and benefit from treatment as usual and which populations may require tailored services to reduce disparities in care.

## 5. Conclusions

Overall, the current study demonstrates that it is feasible to provide care to nurses at their place of employment as part of their healthcare benefits package, though some barriers to mental healthcare utilization remain. Given the prevalence of burnout and work-related stress among nurses, and associated psychiatric distress [3,4,5,6,7,8,9,10], this is an important step toward meeting the mental health needs of an essential group of healthcare workers. Based on these findings, we recommend ongoing investigation of this type of service offering as well as other treatment innovations aiming to improve the accessibility and effectiveness of mental health services for nurses.

## Figures and Tables

**Table 1:** Sample demographics.

Variable	n	%
Gender Identity		
Cisgender woman	110	85.3
Cisgender man	14	10.9
Unknown/declined to answer	5	3.8
Race		
Asian	18	14.0
Black	22	17.1
Multiracial	4	3.1
Native American	2	1.6
White	75	58.1
Unknown/declined to answer	8	6.1
Ethnicity		
Hispanic/Latino	24	18.6
Not Hispanic/Latino	87	67.4
Unknown/declined to answer	18	14.0
Sexual Orientation		
Heterosexual	111	86.0
Gay/lesbian	7	5.4
Bisexual	1	0.8
“I don’t know”	1	0.8
Unknown/declined to answer	9	7.0
Age	M = 38.47	Range = 23–65

**Table 2:** Presenting mental health problems.

Diagnosis	n	%
Depressive disorder	55	48.2
Anxiety disorder	50	43.9
PTSD or trauma/stressor-related disorder	22	19.3
Adjustment disorder	20	17.5
Substance use disorder	2	1.8
Bipolar spectrum disorder	1	0.6
Obsessive-compulsive and related disorder	1	0.6
Personality disorder	1	0.6
Anorexia nervosa	1	0.6
Work-related Stressor	n	%
Identified as contributing to distress	69	53.5

*Note.* Total adds up to greater than 100% for diagnosis as some patients presented with multiple diagnoses.

**Table 3:** Service utilization by service type.

	Medication Management	Individual Therapy	Group Therapy	Couples Therapy	Medication + Therapy
Number of patients	76 ^a^	88 ^b^	12 ^c^	8 ^d^	52
Scheduled appointments (M, SD)	15.9 (18.6)	19.8 (19.6)	19.1 (18.7)	26.5 (32.2)	47.9 (36.9)
Completed appointments (M, SD)	11.7 (15.1)	15.3 (15.7)	8.1 (7.1)	20.9 (22.7)	35.2 (27.9)
Cancelled appointments (M, SD)	3.0 (3.3)	2.5 (3.3)	9.8 (14.6)	2.5 (2.8)	8.3 (10.6)
No showed appointments (M, SD)	0.8 (1.4)	1.9 (3.3)	1.2 (1.4)	3.1 (7.3)	4.2 (5.6)
Attendance rate (%)	73.5 (19.5)	76.6 (22.0)	47.9 (25.0)	85.3 (16.2)	74.7 (15.7)

^a–d^ Includes patients who may have participated in more than one service.

**Table 4:** Suggested mental health programming for hospital-based nursing staff.

Prevention Efforts
Provide psychoeducation on the signs, symptoms, and treatment offerings for all nursing staff during onboarding to increase awareness and reduce stigma.
Train managers to identify signs of mental ill health among their staff and appropriately refer for care.
Encourage employee participation in pro-mental health activities, such as Mental Health Month and seminars on mental health, to reduce stigma. Survey nurses on the support needed and preferences for care.
**Intervention Efforts**
Include mental healthcare as a covered option in employee insurance plans.
Provide covered leave time to attend mental health appointments during the workday.
Offer on-site mental health services (e.g., medication management, psychotherapy) with in-person and virtual appointment options for employees.
Develop unit-based outreach programs to provide psychoeducation and support for mental health to reduce stigma, create cultures of wellness, and provide accessible support.

## Data Availability

The dataset presented in this article is not available due to privacy considerations.
